# Detecting central fixation by means of artificial neural networks in a pediatric vision screener using retinal birefringence scanning

**DOI:** 10.1186/s12938-017-0339-6

**Published:** 2017-04-27

**Authors:** Boris I. Gramatikov

**Affiliations:** Laboratory of Ophthalmic Instrument Development, The Krieger Children’s Eye Center at the Wilmer Institute, Wilmer Eye Institute, 233, The Johns Hopkins University School of Medicine, 600 N. Wolfe Street, Baltimore, MD 21287-9028 USA

**Keywords:** Artificial neural networks, Vision screener, Amblyopia, Strabismus, Fixation detection, Birefringence, Retina

## Abstract

**Background:**

Reliable detection of central fixation and eye alignment is essential in the diagnosis of amblyopia (“lazy eye”), which can lead to blindness. Our lab has developed and reported earlier a pediatric vision screener that performs scanning of the retina around the fovea and analyzes changes in the polarization state of light as the scan progresses. Depending on the direction of gaze and the instrument design, the screener produces several signal frequencies that can be utilized in the detection of central fixation. The objective of this study was to compare artificial neural networks with classical statistical methods, with respect to their ability to detect central fixation reliably.

**Methods:**

A classical feedforward, pattern recognition, two-layer neural network architecture was used, consisting of one hidden layer and one output layer. The network has four inputs, representing normalized spectral powers at four signal frequencies generated during retinal birefringence scanning. The hidden layer contains four neurons. The output suggests presence or absence of central fixation. Backpropagation was used to train the network, using the gradient descent algorithm and the cross-entropy error as the performance function. The network was trained, validated and tested on a set of controlled calibration data obtained from 600 measurements from ten eyes in a previous study, and was additionally tested on a clinical set of 78 eyes, independently diagnosed by an ophthalmologist.

**Results:**

In the first part of this study, a neural network was designed around the calibration set. With a proper architecture and training, the network provided performance that was comparable to classical statistical methods, allowing perfect separation between the central and paracentral fixation data, with both the sensitivity and the specificity of the instrument being 100%. In the second part of the study, the neural network was applied to the clinical data. It allowed reliable separation between normal subjects and affected subjects, its accuracy again matching that of the statistical methods.

**Conclusion:**

With a proper choice of a neural network architecture and a good, uncontaminated training data set, the artificial neural network can be an efficient classification tool for detecting central fixation based on retinal birefringence scanning.

## Background

Amblyopia (“lazy eye”) is poor development of vision from prolonged suppression in an otherwise normal eye, and is a major public health problem, with impairment estimated to afflict up to 3.6% of children—and more in medically underserved populations [1]. Reliable detection of eye alignment with central fixation (CF) is essential in the diagnosis of amblyopia. Further, there is a need for a commercially available and widely accepted automated screening instrument that can reliably detect strabismus and defocus in young subjects [2]. Our laboratory has been developing novel technologies for detecting accurate eye alignment directly, by exploiting the birefringence (a property that changes the polarization state of light) of the uniquely arranged nerve fibers (Henle fibers) surrounding the fovea. We employed retinal birefringence scanning (RBS), a technique that uses the changes in the polarization of light returning from the eye, to detect the projection into space of the array of Henle fibers surrounding the fovea [3–5]. In RBS, polarized near-infrared light is directed onto the retina in a circular scan, with a fixation point in the center, and the polarization-related changes in light retro-reflected from the ocular fundus are analyzed by means of differential polarization detection. Due to the radially symmetric arrangement of the birefringent Henle fibers, a characteristic frequency appears in the obtained periodic signal when the scan is centered on the fovea, indicating central fixation. By analyzing frequencies in the RBS signal from both eyes simultaneously, the goodness of eye alignment can be measured, and thus strabismus (misaligned eyes) can be detected. RBS technology is the only known technology that can detect central fixation remotely using true anatomical information (position of the fovea). An early version of the “pediatric vision screener” (PVS) was designed in our lab and then tested at the Boston Children’s Hospital, [6–10]. This prototype device has been developed into a commercial instrument that detects eye alignment (REBIScan, Boston, MA, USA).

Meanwhile, development of the RBS technology has continued in our lab, resulting in a series of central fixation detecting devices with no moving parts [11, 12], devices for *continuous* monitoring of fixation [13], a device for biometric purposes [14], and ultimately an improved PVS that combines “wave-plate-enhanced RBS” [15], or “polarization-modulated RBS” [16, 17], for detecting strabismus, with added technology for assessing proper focus of both eyes simultaneously. Polarization-modulated RBS is an optimized upgrade of RBS, based upon our theoretical and experimental research and computer modeling, using a spinning half wave plate (HWP) and a fixed wave plate (WP) to yield high and uniform signals across the entire population. In addition, using a technique named “phase-shift-subtraction” (PhSS), the new PVS eliminated the need for initial background measurement [15–17].

Depending on the direction of gaze and the design of the instrument, the screener produces several signal frequencies that can be utilized in the detection of central fixation. Using a computer model involving all polarization-changing components of the system, including the Henle fibers and the cornea, we found that by spinning the HWP 9/16-ths as fast as the circular scan, strong signals are generated that are odd multiples of half of the scanning frequency [17]. With central fixation, two frequency components predominate the RBS signal: 2.5 or 6.5 times the scanning frequency *f*_s_, depending on the corneal birefringence. With paracentral fixation, these frequencies practically disappear, being replaced by 3.5 *f*_s_ and 5.5 *f*_s_. Therefore, the relative strengths of these four frequency components in the RBS signal distinguishes between central and paracentral fixation. In addition, a strong, spin-generated, 4.5 *f*_*s*_ frequency in our RBS signal that is practically independent of corneal birefringence and of the position of the scanning circle with respect to the center of the fovea [16]. This “spin-generated frequency” is thus well suited for normalization of the signal, in order to limit the subject-to-subject variability.

The PVS instrument design has been described in detail elsewhere, and encouraging results have been reported [16–18]. We validated the performance of this research instrument on an initial group of young test subjects—18 patients with known vision abnormalities (8 male and 10 female), ages 4–25 (only one above 18), and 19 control subjects with proven lack of vision issues. Four statistical methods were used to derive decision making rules that would best separate patients with abnormalities from controls. Method 1 (termed “Simple threshold”) employed gradual changing of an adaptive threshold *θ* for the normalized combined power at CF frequencies (*P*_*2.5*_ + *P*_*6.5*_)/*P*_*4.5*_, in order to minimize the classification errors. Methods 2, 3 and 4 employed linear discriminant analysis, basically using a linear combination of respectively 2, 3 or 4 features (in our case normalized signal powers at different frequencies) to separate the two classes (CF vs para-CF). Ultimately, classification is based on a linear classifier involving the coefficients of a 2-, 3- or 4-way discriminant function. Sensitivity and specificity were calculated for each method [18]. The discriminant functions methods provided excellent specificity of 100%, but relatively low sensitivities of 90% or below. This meant that although all detected abnormalities would be true, at least 10% of the children with strabismus would be missed. For this reason we chose the “Simple threshold” (Method 1) which on the calibration data gave sensitivity of 99.17% and specificity of 96.25%.

The objective of the present study was, based on the characteristic signal frequencies mentioned above, to develop and test an artificial neural network (ANN) for the detection of central fixation, and to compare it with the classical statistical methods, reported earlier.

Artificial neural networks have quite often been used for diagnostic purposes in the past two–three decades. Applications include the diagnosis of myocardial infarction [19], waveform analysis of biomedical signals, medical imaging analysis and outcome prediction [20], automatic detection of diabetic retinopathy [21], nephritis and heart disease [22], biochemical data and heart sounds for valve diagnostics [23], and many more. There are reports in the literature relating to the use of artificial neural networks for eye tracking [24–28]. They are mostly used as part of a human–computer interface, or as an aid for the handicapped, and work typically with a camera which tracks the pupil using either infrared or visible light images of the pupil. Through proper training, neural networks can provide precise individual calibration. Such networks often employ on the order of 20–200 dimensions (hidden neurons), and can require significant computing time. They are accurate to approximately 0.75°. In our application, the signals are available as spectral powers at just a few frequencies, generated upon retinal birefringence scanning around the fovea. They allow the detection of central fixation with much higher precision (0.1°) without the need for full-range eye tracking or calibration, while allowing some head mobility. Applications of artificial neural networks for this purpose are unknown to the author.

## Methods

The optics, electronics, and signal analysis of the PVS have been reported in more detail previously [16–18]. This present work focuses on the use of artificial neural networks as an alternative to classical statistical methods. The goal of this study was, if possible, to improve the classification algorithms, as well as to validate the performance of the research instrument on the same group of young test subjects that was used in the previous study, with the addition of two more subjects. All subjects’ data were analysed with both the methods from the previous paper, and the neural networks method reported here. For more detail on the human subject data, please see the “Data” section below. The neural network performance is compared with the four statistical methods that were applied to the same dataset earlier, and the ability to separate patients with abnormalities from controls was investigated.

### Artificial neural networks

Artificial neural networks have been widely used, and the related theory has matured in the last three decades [29–35]. Feedforward neural networks (FNNs) are static, i.e. networks with no feedback elements and no delays. They are widely used to solve complex problems in pattern classification, system modeling and identification, and non-linear signal processing, and in analyzing non-liner multivariate data. One of the characteristics of the FNN is its learning (or training) ability [36]. It has a learning process in both hidden and output layers. By training, the FFNs can give correct answers not only for learned examples, but also for the models similar to the learned examples, showing their strong associative ability and rational ability which are suitable for solving large, nonlinear, and complex classification and function approximation problems. The classical method for training FNNs is the backpropagation (BP) algorithm [31] which is based on the gradient descent optimization technique.

Many tools have been developed for creating and testing ANN networks. Among them, probably the most significant one is the *Neural Networks Toolbox* for MATLAB from MathWorks, Inc. [37] The author employed this toolbox for creating, training, and testing the network with both calibration and clinical data. Another useful tool widely used in the field is the *Netlab* simulation software, designed to provide the central tools necessary for the simulation of theoretically well-founded neural network algorithms for use in teaching, research, and applications development. It consists of a library of MATLAB functions and scripts based on the approach and techniques described in the book *Neural Networks for Pattern Recognition* by Dr. Christopher Bishop [38], Department of Computer Science and Applied Mathematics at Aston University, Birmingham, UK.

### Neural network architecture

**Fig. 1:**
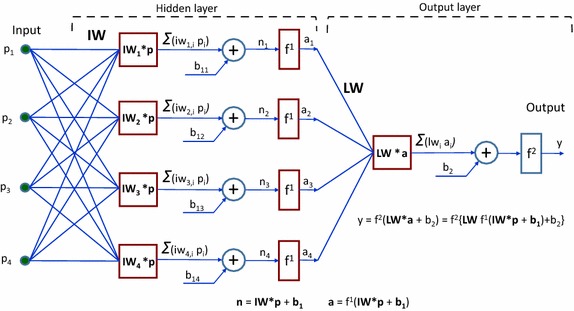
Neural network architecture. A two-layer architecture has been employed, consisting of one hidden layer and one output layer. The network has an input **p** containing four inputs (p_1_–p_4_), representing the four normalized RBS spectral powers, respectively P_2.5_/P_4.5_, P_3.5_/P_4.5_, P_5.5_/P_4.5_, and P_6.5_/P_4.5_. The hidden layer contains four neurons. The output of the net signals presence or absence of central fixation

For the present application, several ANN architectures were tested. To avoid overfitting (explained later under Generalization), a relatively simple architecture was selected (Fig. 1), consisting of one hidden layer and one output layer. This two-layer network has an input **p** containing four inputs (p_1_–p_4_), representing the normalized RBS spectral powers, respectively P_2.5_/P_4.5_, P_3.5_/P_4.5_, P_5.5_/P_4.5_, and P_6.5_/P_4.5_, that are generated during retinal birefringence scanning. The hidden layer contains four neurons. Each neuron is connected to each of the inputs through the input weight matrix **IW**:

1 $${\mathbf{IW}} = \left[ {\begin{array}{*{20}c} {iw_{1,1} } & \cdots & {iw_{1,4} } \\ \vdots & \ddots & \vdots \\ {iw_{4,1} } & \cdots & {iw_{4,4} } \\ \end{array} } \right]$$

The i-th neuron has a summer that gathers its weighted inputs iw_i,j_ and a bias b_1,i_, to form its scalar output n_i_ as:

2 $${\text{n}}_{\text{i}} = {\text{ iw}}_{{{\text{i}}, 1}} {\text{p}}_{ 1} + {\text{ iw}}_{\text{i,2}} {\text{p}}_{ 2} + {\text{ iw}}_{{{\text{i}}, 3}} {\text{p}}_{ 3} + {\text{ iw}}_{{{\text{i}}, 4}} {\text{p}}_{ 4} + {\text{ b}}_{{ 1,{\text{i}}}}$$

equivalent to a dot-product (inner product):

3 $${\mathbf{n}} = {\mathbf{IW}}*{\mathbf{p}} + \varvec{b}_{1}$$

where **b**_**1**_ is a four-element vector representing the four biases, one for each neuron.

Each n_i_ then is processed by a sigmoid transfer function f^1^ to deliver a neuron output a_i_. The 4-element output vector of the four neurons (and the hidden layer as a whole) can be represented in matrix form as:

4 $${\mathbf{a}} = {\text{f}}^{1} ({\mathbf{IW}}*{\mathbf{p}} + \varvec{b}_{1} )$$

The four neuron outputs are then fed to the output layer, which has a neuronal structure as well. Its scalar output y can be represented by the equation:

5 $${\text{y }} = {\text{ f}}^{ 2} \left( {{\mathbf{LW}}*{\mathbf{a}} + {\text{ b}}_{ 2} } \right) \, = {\text{ f}}^{ 2} \left\{ {{\mathbf{LW}}{\text{ f}}^{ 1} \left( {{\mathbf{IW}}*{\mathbf{p}} + {\mathbf{b}}_{{\mathbf{1}}} } \right) + {\text{b}}_{ 2} } \right\}$$

where **LW** is the output layer weight matrix and the scalar b_2_ is the output neuron’s bias:

6 $${\mathbf{LW}} = \, \left[ {{\text{lw}}_{ 1} {\text{ lw}}_{ 2} {\text{ lw}}_{ 3} {\text{ lw}}_{ 4} } \right]$$

The weights and biases were calculated during the training of the network, as explained later. The sigmoid transfer functions for the hidden layer f^1^ and for the output layer f^2^ were chosen to be the same, namely of type Log-Sigmoid transfer function (logsig):

7 $$\tt{logsig}\left( \texttt{{n}} \right)= \texttt{1}/\left( {\text{\tt{1 + exp}}\left( { - {\texttt{n}}} \right)} \right)$$

The function logsig generates outputs between 0 and 1 as the neuron’s net input goes from negative to positive infinity. As mentioned above, the neural network shown in Fig. 1 is a FFN. Feedforward networks consist of a series of layers. The first layer has a connection from the network input. Each subsequent layer has a connection from the previous layer. The final layer produces the network’s output. FFNs can be used for any kind of input to output mapping. A feedforward network with one hidden layer, and enough neurons in the hidden layers, can fit any finite input–output mapping problem. It can be used as a general function approximator. It can approximate, arbitrarily well, any function with a finite number of discontinuities, given sufficient a number of neurons in the hidden layer. Specialized versions of the feedforward network include fitting and pattern recognition networks. The pattern recognition networks are the ANN of choice when solving classification problems, such as ours. In pattern recognition problems, we want a neural network to classify inputs into a set of target categories. Thus, pattern recognition networks are FFNs that can be trained to classify inputs according to already verified *target* classes, in our case verified central fixation versus paracentral fixation.

### Creating the neural network

Following the above reasoning, our neural network was created (as a network object) using the MATLAB Toolbox’s pattern recognition network creation function patternnet:

8 $${\texttt{net} = \tt{patternnet}}\left( {{\texttt{hiddenLayerSize}, \tt{trainFcn}}} \right);$$

The parameter hiddenLayerSize here is 4, corresponding to the four neurons in the hidden layer. One can change the number of neurons if the network does not perform well after training, and then retrain. The parameter trainFcn defines the training function, which in our case is ‘trainscg’, standing for the *scaled conjugate gradient backpropagation* method for updating weight and bias values during training [39]. It performed slightly better on our data than the popular and faster Levenberg–Marquardt (LM) training algorithm [40]. Backpropagation (explained below) is used to calculate derivatives of performance perf with respect to the weight and bias variables.

### Data

To define a pattern recognition problem, data is generally arranged in a set of Q input vectors (measurements) as columns in a matrix. Then another set of Q target vectors is arranged, so that they indicate the classes to which the input vectors are assigned. Classification problems involving only two classes (as in our case) can be represented by target vectors consisting of either scalar 1/0 elements, which is the format used in this study. Alternatively, the target could be represented by two-element vectors, with one element being 1 and the other element being 0. In the general case, the target data for pattern recognition networks should consist of vectors of all zero values except for a 1 in element *i*, where *i* is the class they represent.

The **calibration data** were comprised of the same data set that was used in an earlier study [18]. Briefly, with the pediatric vision screener [16–18] we recorded signals from five asymptomatic normal volunteers, ages 10, 18, 24, 29 and 39, two female and three male, of them three Caucasian, one African American, and one Asian, all properly consented. The subjects were asked to look first at the blinking target in the center of the scanning circle, for central fixation (CF). Twelve measurements of duration 1 s were taken in order to obtain representative data while taking into consideration also factors like fixation instability and distractibility. The calculated FFT powers for each measurement were saved on disk. The same type of measurement was repeated with each of the subjects looking at imaginary “targets” on the scanning circle (1.5° off center) at 12, 3, 6, and 9o’clock. The spacing was chosen such that there would be a sufficient distance between the targets, to avoid confusion in the test subject, and to overcome the natural instability of fixation. More fixation points or more than 12 measurements per target have proven to diminish the efficiency of data collection, because of fatigue occurring in the test subjects. The data, consisting of powers *P*_*2.5*_ *, P*_*6.5*_ *, P*_*3.5*_ *, P*_*5.5*_ and *P*_*4.5*_ for each of the 12 measurements of each eye of all five test subjects, were bundled into two groups: a group for central fixation (120 “eyes,” the “CF set”) and a group for paracentral fixation (480 “eyes,” the “para-CF set”). Data from these two controlled groups were used to create and calibrate the ANN. The data were organized as an input matrix of 4 rows and Q columns, with Q = 600 (120 measurements with CF and 480 measurements with para-CF). The target vector was a vector of length Q = 600, each element of which was either 1 (CF) or 0 (para-CF). These inputs and targets were used for training, validation and testing the network. One can reasonably argue that this number of subjects (5) and eyes (10) is insufficient for providing reliable calibration with regard to the two classes (CF versus para-CF). Yet, the variability of the RBS signals’ waveforms (and respectively the five derived frequency powers) depend to a much higher extent on the subject’s direction of gaze and the ability to fixate, than on the individual variability of the foveal and corneal birefringence. This invariability, especially to corneal birefringence, was achieved with the new design, as reported in our previous work [15–17]. The birefringence of the fovea is largely constant. It is the corneal birefringence that affects the signals. The cornea, acting as a retarder of a certain retardance and azimuth, influences the orientation of the polarization cross. In the design of the PVS [18], the corneal birefringence was compensated for by means of a wave plate (retarder), achieving broad uniformity across the population studied. The wave plate was optimized by means of a dynamic computer model of the retinal birefringence scanning system (including the retina and the cornea as part of a train of optical retarders) and based on the data from a database of 300 eyes [15].

The **clinical** data were also obtained with the pediatric vision screener (following an institutionally approved IRB protocol), and were almost identical with the data set used in [18], with the addition of just two more subjects, both of whom were independently verified by a pediatric ophthalmologist. Thus, the total was 39 test subjects: 19 properly consented patients with known abnormalities (9 male and 10 female, of which 12 Caucasian, 2 African American, and 5 Asian), ages 4–25 (only one above 18), and 20 control subjects with proven lack of vision issues (10 male and 10 female, of which 16 Caucasian, 1 African American, and 3 Asian), ages 2–37 (only 4 above 18), all properly consented. All were recruited from the patients of the Division of Pediatric Ophthalmology at the Wilmer Eye Institute, or the patients’ siblings. All subjects underwent a vision exam by an ophthalmologist, during which eye alignment and refraction were tested. Eye alignment was tested by means of the *cover test*. First the unilateral cover test was performed. During the unilateral cover test, the patient is asked to focus on a distant object while the doctor covers each of the eyes in turn. If either of the uncovered eyes has to move to focus on the object, this may be evidence of strabismus. The second part of the exam is the alternating cover test. The patient is asked to focus on an object while the eye cover is switched from one eye to the other. If the doctor detects eye movement after the eye cover is removed, this is an indication of phoria (tendency to deviation of the eyes from the normal when fusional stimuli are absent). A significant amount of phoria can lead to eyestrain and/or double vision. On the whole, verified information was available from a total of 78 eyes. Data were organized as an input matrix of 4 rows and Q columns (Q = 78 eyes). The target vector was a vector of length Q (Q = 78), each element of which was either 1 (CF) or 0 (para-CF). These inputs and targets, as well as the network outputs, were used for testing the performance of the ANN and comparing it to the statistical methods reported earlier [18].

### Preprocessing and postprocessing

Neural network training can be made more efficient if one performs certain preprocessing steps on the network inputs and targets [35, 37]. The sigmoid transfer functions that are generally used in the hidden layers become essentially saturated when the net input is greater than three. If this happens at the beginning of the training process, the gradients will be very small, and the network training will be very slow. It is standard practice to normalize the inputs before applying them to the network. Generally, the normalization step is applied to both the input vectors and the target vectors in the data set. The input processing functions used here are removeconstantrows (removes the rows of the input vector that correspond to input elements that always have the same value, because these input elements are not providing any useful information to the network), and mapminmax (normalize inputs/targets to fall in the range [−1,1]). For outputs, the same processing functions (removeconstantrows and mapminmax) are used. Output processing functions are used to transform user-provided target vectors for network use. Then, network outputs are reverse-processed using the same functions to produce output data with the same characteristics as the original user-provided targets.

### Dividing the data

When training multilayer networks, the general practice is to first divide the data into three subsets. The first subset is the *training set*, which is used for computing the gradient and updating the network weights and biases. This set is presented to the network during training, and the network is adjusted according to its error. The second subset is the *validation set*. It is used to measure network generalization, and to halt training when generalization stops improving. The error on the validation set is monitored during the training process. The validation error normally decreases during the initial phase of training, as does the training set error. However, when the network begins to overfit the data, the error on the validation set typically begins to rise. The network weights and biases are saved at the minimum of the validation set error. The *test set* has no effect on training and so provides an independent measure of network performance during and after training. Test set error is not used during training, but it is used to compare different models. It is also useful to plot the test set error during the training process. If the error on the test set reaches a minimum at a significantly different iteration number than the validation set error, this might indicate a poor division of the data set.

In the *Neural Networks Toolbox* for MATLAB [37], there are four functions provided for dividing data into training, validation, and test sets. They are dividerand (divide data randomly, the default), divideblock (divide into contiguous blocks), divideint (use interleaved selection), and divideind (divide by index). The data division is normally performed automatically when the network is trained. In this study, the dividerand function was used, with 70% of the data randomly assigned to training, 15% of the data randomly assigned to validation, and 15% of the data randomly assigned to the test set. This is the default partitioning in *Neural Networks Toolbox*. The appropriateness of this division is discussed in the “Discussion and limitations” below.

### Initializing weights (init)

Before training a feedforward network, one must initialize the weights and biases. The configure command automatically initializes the weights, but one might want to reinitialize them. This is done with the init command. This function takes a network object as input and returns a network object with all weights and biases initialized. Here is how a network is initialized (or reinitialized):

9 $$\text{\tt{net} = \tt{init}}\left( {\text{\tt{net}}} \right)\text{;}$$

### Performance function

Once the network weights and biases are initialized, the network is ready for training. The training process requires a set of examples of proper network behavior—network inputs p and target outputs t. The process of training a neural network involves tuning the values of the weights and biases of the network to optimize network performance, as defined by the network performance function. The default performance function for feedforward networks is mean square error mse-the average squared error between the network outputs y and the target outputs t [37]. It is defined as follows:

10 $$F = mse = \frac{1}{N}\mathop \sum \limits_{i = 1}^{N} \left( {e_{i} } \right)^{2} = \frac{1}{N}\mathop \sum \limits_{i = 1}^{N} \left( {t_{i} + y_{i} } \right)^{2}$$

For a neural network classifier, during training one can use mean squared error or cross-entropy error, with cross-entropy error being considered slightly better [41]. We tested both methods on a subset of the data, and obtained slightly better results with the cross-entropy method. Which is why the network performance evaluation in this study was done by means of the cross-entropy method:

11 $${\texttt{net.performFcn}} = \lq\texttt{crossentropy}\hbox{'};$$

The MATLAB performance function has the following format:

12 $${\tt{perf}} = \tt{crossentropy}\left( \tt{net},\tt{targets},\tt{outputs},\tt{perfWeights} \right)$$

It calculates a network performance given targets (t) and outputs (y), with optional performance weights and other parameters. The function returns a result that heavily penalizes outputs that are extremely inaccurate (y near 1−t), with very little penalty for fairly correct classifications (y near t). Minimizing cross-entropy leads to good classifiers. The cross-entropy for each pair of output-target elements is calculated as:

13 $$\tt{ce} = {-}\tt{t}\; \tt{.^*} \;\tt{log} ( {\tt{y}} )$$

where .* denotes element-by-element multiplication. The aggregate cross-entropy performance is the mean of the individual values:

14 $${\tt{perf}} = \tt{sum}( {\tt{ce}} ( {\text{:}} ) )\tt{/numel} ( {\tt{ce}} )$$

In the special case of N = 1 (our case) when the output consists of only one element (y), the outputs and targets are interpreted as binary encoding. That is, there are two classes with targets of 0 and 1. The binary cross-entropy expression is:

15 $$\tt{ce} = {-}\tt{t}\; {\tt{.^*\;log}}\left( {\tt{y}} \right) - \left( {\tt{1 - t}} \right)\text{ }{\tt{.^* log}}\left( {\tt{1 - y}} \right)$$

where .* denotes element-by-element multiplication.

### Training the network

For training multilayer feedforward networks, any standard numerical optimization algorithm can be used to optimize the performance function, but there are a few key ones that have shown excellent performance for neural network training. These optimization methods use either the gradient of the network performance with respect to the network weights, or the Jacobian of the network errors with respect to the weights. The gradient and the Jacobian are calculated using a technique called the *backpropagation* algorithm, which involves performing computations backward through the network. The backpropagation computation is derived using the chain rule of calculus and is described in more detail in [33] and in [31]. As a note on terminology, the term “backpropagation” is sometimes used to refer specifically to the gradient descent algorithm, when applied to neural network training. That terminology is not used here, since the process of computing the gradient and Jacobian by performing calculations backward through the network is applied in all of the training functions offered by MATLAB’s Neural Networks Toolbox. It is clearer to use the name of the specific optimization algorithm that is being used (i.e. ‘trainscg’, ‘trainlm’, ‘trainbr’, etc.), rather than to use the term backpropagation alone.

Neural networks can be classified into static and dynamic categories. Static networks (which are essentially the FFNs) have no feedback elements and contain no delays; the output is calculated directly from the input through feedforward connections. In dynamic networks, the output depends not only on the current input to the network, but also on the current or previous inputs, outputs, or states of the network. These dynamic networks may be recurrent networks with feedback connections or feedforward networks with imbedded tapped delay lines (or a hybrid of the two) [34]. For static networks, the backpropagation algorithm is usually used to compute the gradient of the error function with respect to the network weights, which is needed for gradient-based training algorithms [42].

The actual training was completed using the function from MATLAB’s Neural Networks Toolbox:

16 $$\left[ {\text{\tt{net,tr}}} \right]\text{ \tt{= train}}\left( {\text{\tt{net,x,t}}} \right)$$

with x being the input matrix (600 column vectors of size x), and t being the target vector of size 600 (total number of observations in the calibration set).

Network performance was calculated using the perform function

17 $$\text{\tt{performance = perform}}\left( {\text{\tt{net,t,y}}} \right)$$

which takes the network object, the targets t and the outputs y and returns performance using the network’s performance function net.performFcn (crossentropy in our case). Note that training automatically stops when generalization stops improving, as indicated by an increase in the cross-entropy error of the validation samples.

### Generalization

Neural networks are sensitive to the number of neurons in their hidden layers. Too few neurons can lead to underfitting. Too many neurons can contribute to overfitting, in which all training points are well fitted, but the fitting curve oscillates significantly between these points, and so do the calculated coefficients. In ANN terms, the model does not *generalize* well. It is apparent from testing with an increasing complexity that as the number of connections in the network increases, so does the propensity to overfit to the data. The phenomenon of overfitting can always be seen as we make our neural networks deep (complex).

In this study, the number of neurons in the hidden layer was chosen empirically. On the clinical data, less than four input neurons did not provide the accuracy achieved with 4-8 neurons in the hidden layer, most likely because of underfitting. At about 10 neurons and upwards, the accuracy started to decrease again, because of overfitting. The choice of 4 hidden neurons was made for two reasons: (a) keep the network generalized (i.e. to avoid overfitting), and (b) to keep it simple and computationally fast. With respect to the number of hidden layers, no significant improvement was achieved with a two-hidden-layer structure, regardless of the number of neurons in each layer.

MathWorks suggests several ways to improve network generalization and avoid overfitting [37, 43]. One method for improving network generalization is to use a network that is just large enough to provide an adequate fit. The larger network we use, the more complex the functions the network can create. If a small enough network is used, it will not have enough power to overfit the data. One can check the *Neural Network Design* example nnd11gn in [33] to investigate how reducing the size of a network can prevent overfitting. Another approach is retraining. Typically each backpropagation training session starts with different initial weights and biases, and different divisions of data into training, validation, and test sets. These different conditions can lead to quite different solutions for the same problem. Therefore, it is a good idea to train several networks, in order to ensure that a network with good generalization is found.

The default method for improving generalization is the so-called *early stopping*. This technique is automatically provided for all of the supervised network creation functions in the Neural Networks toolbox, including the backpropagation network creation functions such as feedforwardnet and patternnet. As explained before, in this technique the available data are divided into three subsets. The first subset is the training set, which is used for computing the gradient and updating the network weights and biases. The second subset is the validation set. The error on the validation set is monitored during the training process. The validation error normally decreases during the initial phase of training, as does the training set error. However, when the network begins to overfit the data, the error on the validation set typically begins to rise. When the validation error increases for a specified number of iterations (net.trainParam.max_fail), the training is stopped, and the weights and biases at the minimum of the validation error are returned. The test set error is not used during training, but it is used to compare different models. It is also useful to plot the test set error during the training process. If the error in the test set reaches a minimum at a significantly different iteration number than the validation set error, this might indicate a poor division of the data set [43].

There is yet another method for improving generalization, called *regularization*. It involves modifying the performance function, which is normally chosen (mse, cross-entropy, or other). Using a modified performance function causes the network to have smaller weights and biases, forcing the network response to be smoother and less likely to overfit [43]. Regularization can be done automatically by using the Bayesian regularization training function trainbr. This can be done by setting net.trainFcn to ‘trainbr’. This will also automatically move any data in the validation set to the training set [37].

## Results

### Network creation and training

**Fig. 2:**
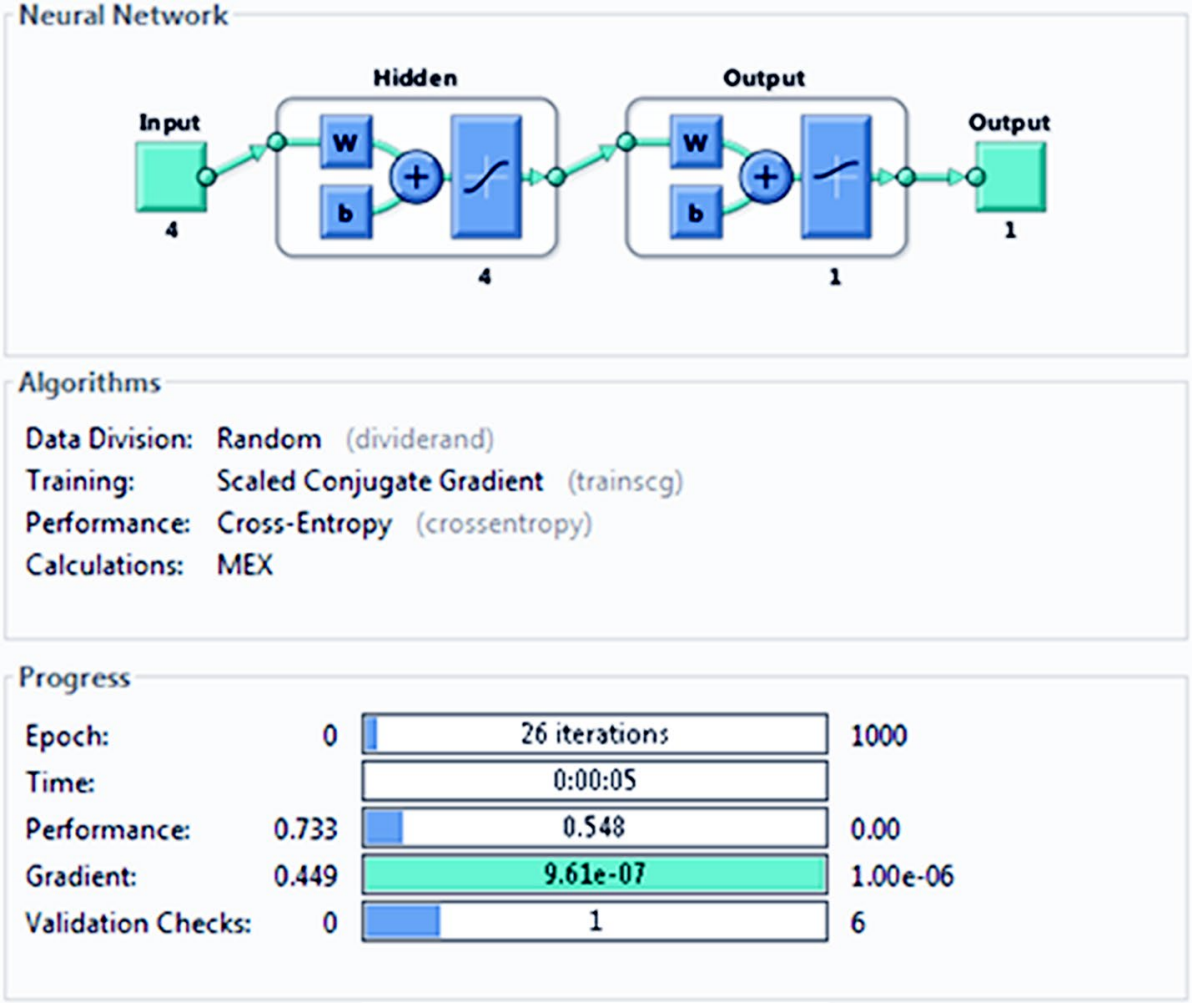
Neural network training tool (nntraintool) representing the training process. The upper part illustrates the network architecture, as shown in Fig. 1, this time generated by MATLAB. Training was stopped after iteration 26, at performance 0.548

**Fig. 3:**
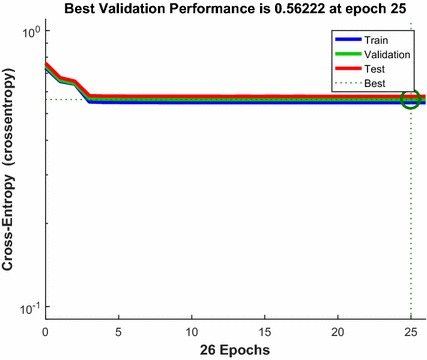
Validation performance, based on the cross-entropy error. Minimizing cross-entropy results in good classification. Lower values are better. Zero means no error

**Fig. 4:**
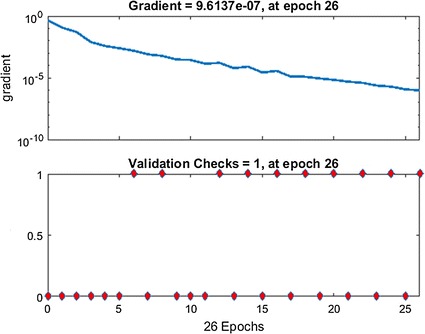
Dynamics of the neural network training state in terms of gradient of the cross-entropy, on a logarithmic scale. At the endpoint, the gradient was 9.6137 × 10^−7^

**Fig. 5:**
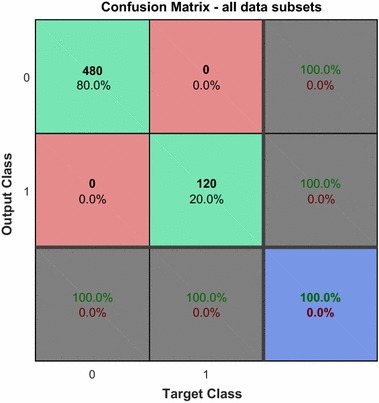
The combined confusion matrix for the three kinds of data (train, validate, test) from the calibration set. The *green diagonal* cells show the number and percentage of correct classifications by the trained network. The *red diagonal* represents the incorrect classifications, which are 0 for each target class and for each output class. The *lower right blue square* illustrates the overall accuracy

The neural network was trained, validated, and tested on the calibration data of 600 eyes (explained in more detail under the “Data” subsection in “Methods” above). Figure 2 shows the NN training process (nntraintool). The upper part illustrates the network architecture, as shown in Fig. 1, this time generated by MATLAB. The tool shows the algorithms used, as well as the training progress. Training was stopped after iteration 26, at performance 0.548. The performance graph is presented in Fig. 3, showing how cross-entropy is minimized for good classification. Before epoch 26, the best validation performance of 0.562 was reached at epoch 25, which is only slightly higher than the final 0.548. Figure 4 shows the dynamics of the training state in terms of gradient of the cross-entropy, on a logarithmic scale. At the endpoint, the gradient was 9.6137 × 10^−7^, which can be considered a good value at which to stop for this set of data. The combined confusion matrix for the three kinds of data (train, validate, test) from the calibration set is presented in Fig. 5. In this figure, the first two diagonal cells (in green) show the number and percentage of correct classifications by the trained network. For example 480 measurements (in the target set of class 0) are correctly classified as paracentral fixation (output set of class 0). This corresponds to 80.0% of all 600 measurements. Similarly, 120 cases (in the target set of class 1) are correctly classified as central fixation (output set of class 1). This corresponds to 20.0% of all measurements. The other diagonal (red) represent the incorrect classifications, which are 0 for each target class and for each output class. The lower right blue square illustrates the overall accuracy.

Overall, 100.0% of the predictions are correct and there are no wrong classifications. In terms of sensitivity and specificity, this corresponds to *sensitivity* = 100.0% and *specificity* = 100.0% (Table 1), and exceeds the results from our previous study [18], where none of the statistical methods applied to the *same data* reached this accuracy (please see columns CAL in Table 1). The reader should, however, be reminded, that this was achieved at a relatively small size of the training set, with the data having been provided by just 10 eyes from 5 patients.

**Table 1:** Performance of the artificial neural network compared with statistical methods with regard to classifying fixation as central versus paracentral fixation

	Neural network		Simple threshold		Discriminant analysis					
					2D		3D		4D	
	CAL	SBJ	CAL	SBJ	CAL	SBJ	CAL	SBJ	CAL	SBJ
SNS	1.000	0.9851	0.9917	0.9701	0.9083	0.8955	0.9417	0.8358	0.9417	0.8507
SPC	1.000	1.0000	0.9625	1.0000	0.9771	1.0000	1.0000	1.0000	1.0000	1.0000

Data: *CAL* calibration data obtained from 600 controlled measurements from 5 test subjects (12 measurements on both eyes for each target location), *SBJ* clinical data from 39 subjects (78 eyes)

Stats: *SNS* Sensitivity = True Positive/(True Positive + False Negative) = TP/(TP + FN), *SPC* Specificity = True Negative/(True Negative + False Positive) = TN/(TN + FP)

### Weights and biases of the ANN

After initialization and training, the weights for the hidden layer, contained in matrix **IW**, according to (1) above, and as extracted with MATLAB function *cell2mat(net.IW)*, were:

$${\mathbf{IW}} = \left[ {\begin{array}{*{20}c} { 0. 8 3 4 3} & { - 1. 6 7 6 8} & { 0. 3 4 7 6} & { 0. 8 6 9 8} \\ { - 1. 5 3 2 0} & { 1. 3 4 1 7} & { 1. 8 0 4 5} & { - 1. 9 1 9 2} \\ { - 1. 0 2 7 1} & { 0. 6 3 2 9} & { 1. 4 7 5 5} & { - 1. 2 5 0 9} \\ { - 2. 3 1 8 3} & { 0. 6 2 7 0} & { 3. 2 9 6 7} & { - 5. 0 4 8 3} \\ \end{array} } \right]$$

The bias vector for the hidden layer, as accessed with function *cell2mat(net.b(1))*, was

$${\mathbf{b1}} = \begin{array}{*{20}c} {[ - 1. 6 6 7 1} & { - } 0. 1 4 5 7 & { - 0. 0 1 5 5} & { - 2. 3 6 4 8} \\ \end{array} ]$$

The output weights **LW**, as accessed with MATLAB function *cell2mat(net.LW)*, were

$${\mathbf{LW}} = \left[ {\begin{array}{*{20}c} { 1. 5 3 7 9} & { - 2. 9 1 5 3} & { - 1. 8 2 4 5} & { - 1 1. 3 1 0 9} \\ \end{array} } \right]$$

Finally, the output bias, b2 = *cell2mat(net.b(2))*, a scalar, was

b2 = 0.3771

It should be noted that because of the random assignment of the data (training, validation, and test sets), the above weights and coefficients may vary somewhat. This, however, did not impact the sensitivity and specificity significantly. Nevertheless, we trained and retrained the network four times. With all four sessions, both the sensitivity and specificity for the calibration data remained 1.000. For the clinical data, the results from the session which maximized the sensitivity were chosen, because the main goal of this project was to develop a screening device for children, which should not miss lack of central fixation.

Networks with more neurons in the hidden layer, as well as networks with two hidden layers were also tested on the calibration dataset, but they did not improve performance. Their use was avoided because of the risk of potential overfitting.

### Testing the ANN on the clinical data

Once the neural network was created, trained, validated, and tested on the calibration data, in a further step, it was tested on our data set of clinical data (described in detail above under the “Data” subsection in “Methods”) consisting of a subset of strabismic eyes and a control subset of normal eyes, all obtained with the pediatric vision screener. Four normalized spectral powers from a total of 78 eyes were organized as an input matrix of 4 rows and Q columns (Q = 78). The target vector was a vector of length 78, each element of which was either 1 (CF) or 0 (para-CF). The four inputs for each subject were fed to the ANN, and the output was compared each time with the target, which in fact was the doctor’s decision. This allowed the calculation of the sensitivity and specificity of the ANN when applied to the clinical data. Further, these results permitted a comparison between the performance of the ANN and the statistical methods reported earlier [18], such as the simple adaptive threshold that minimized the overall error, or 2-, 3- and 4-way linear discriminant analysis. The results are summarized in Table 1, columns SBJ (human subjects). The two new patients (4 eyes) were quite “tricky” adding two false negative decisions to the “Standard Threshold” method and just one false negative decision to the neural network’s results. Again, the ANN performed slightly better than the other methods, with sensitivity of 0.9851 and specificity of 1.0000, with no false positive decisions and only one false negative decision. Generally, the discriminant-analysis-based methods showed lower sensitivity. Specificity on the clinical data was 1.0000 for all methods except for the 2-way discriminant analysis. Note that the only other method that used all four inputs separately is the 4-way discriminant analysis, giving a sensitivity of 0.9417 for the calibration data, and only 0.8507 for the clinical data. The excellent performance of the ANN is obviously due to the two-layer structure and to the nonlinear (sigmoid) transfer function at the output of each neuron, giving more flexibility, while the performance of all discriminant functions used in the previous study was strictly linear, resembling just one layer of neurons with a linear transfer function, not ideal for pattern recognition.

### Dependence of the classification precision on the training data set

In order to assess the dependence of the classification precision on the selection of the training data set, we ran 10 sessions of calibration followed each time by diagnostic classification. Each time the available calibration data was assigned at random to the training (70%), validation (15%), and test (15%) subsets, as was done with the calibration data whose outcome was presented in Table 1 above. This was performed by means of the following code, executed during each run:

*net*.*divideFcn* *=* ‘*dividerand*’;

*net*.*divideParam*.*trainRatio* *=* *70/100*;

*net*.*divideParam*.*valRatio* *=* *15/100*;

*net*.*divideParam*.*testRatio* *=* *15/100*;

For each run, the sensitivity and specificity of both the calibration and the patient data was calculated, and tabulated in Table 2. The results were somewhat different from the results in the first two columns of Table 1, indicating that the weights of the ANN and the resulting diagnostic precision do depend on the choice of the calibrations subset for training and validation. This also means that a larger calibration pool would have likely given more stable results. At the same time, the sensitivity and specificity did not vary too much, as demonstrated by the standard deviation numbers in the bottom row, indicating that a reasonable diagnostic precision has been reached. The average sensitivity for the clinical data was 0.9806, with the highest sensitivity being 1.0000 and the lowest sensitivity being 0.9552. For diagnostic screening it is the high sensitivity that matters more than high specificity. The former minimizes the number of missed abnormalities, while the latter minimizes the number of subjects falsely referred to the doctor.

**Table 2:** Ten runs of the analysis program (ANN only)

Run	Iterations	CAL		SBJ	
		SNS	SPC	SNS	SPC
1	42	1.0000	1.0000	0.9851	1.0000
2	34	1.0000	1.0000	0.9851	1.0000
3	48	1.0000	1.0000	0.9701	1.0000
4	28	0.9750	1.0000	0.9851	1.0000
5	31	1.0000	1.0000	1.0000	0.9091
6	35	1.0000	1.0000	0.9851	1.0000
7	46	0.9333	1.0000	0.9552	1.0000
8	23	0.9917	0.9938	0.9851	0.9091
9	38	1.0000	1.0000	0.9851	1.0000
10	21	1.0000	0.9958	0.9701	1.0000
Mean→	*35*	*0.9900*	*0.9990*	*0.9806*	*0.9818*
STD→	*9*	*0.0215*	*0.0022*	*0.0123*	*0.0383*

In each run data allocation in the calibration data (600 measurements from 10 eyes from 5 normal subjects) was done at random, while keeping the portions of the training, validation and test sets constant (70, 15, and 15% respectively). Sensitivity (SNS) and specificity (SPC) changed somewhat for both the calibration set *CAL*, and the test subjects set *SBJ* (78 eyes from 39 subjects), depending on the choice of the training set, as did the number of the iterations needed to reach the best validation performance. The performance of the ANN on the test subjects *SBJ* (both patients and controls) is shown in the last two columns

## Discussion and limitations

Although MATLAB was used to create and train the network in this study, porting the code and the ANN weights and biases to another software platform, including an embedded system, is relatively straightforward. This is possible because: (a) the network architecture is known and transparent, (b) the weights and biases are available and can easily be accessed, (c) almost the entire MATLAB code is available as source code, and (d) after modeling in MATLAB and before finalizing the application, the testing can be performed on the target platform with the same data that was used to train the network in MATLAB.

Compared with the 4-way linear discriminant analysis, the ANN with 4 neurons in the hidden layer is more complex, providing its flexibility to differentiate somewhat better between the two classes.

One should acknowledge the statistical limits due to the relatively small number of the learning samples. We used 12 measurements per eye for each direction of gaze, and even though exactly the same 10 eyes from five patients were used for training the ANN, as were used for tuning the statistical methods, it would certainly take a significantly larger study, involving more subjects, to draw a decisive conclusion as to which approach is better. Despite this limitation, the study is a proof of concept of using ANNs for this analysis.

There are several issues that the NN user should have in mind, though. Despite the general success of the backpropagation algorithm, it may generally converge to a **local** minimum, for example, when the mean squared-error objective function (*mse*), or alternatively the cross-entropy is used, and requires a large number of learning iterations to adjust the weights of the FNN. Many attempts have been made to speed up the error BP algorithm. The most well-known algorithms of this type are the conjugate gradient training algorithm [39] (used here), and Levenberg–Marquardt (LM) training algorithm [40]. The computational complexity of the conjugate gradient algorithm (employed here) is heavily dependent on the line search methods. The LM algorithm has a faster speed than gradient training algorithm and hardly gets stuck in a local minimum. It, however, requires much more memory and computational time.

While two-layer feedforward networks can learn virtually any input–output relationship, feedforward networks with more layers might learn complex relationships more quickly. For most problems, it is best to start with two layers, and then increase to three layers, if the performance with two layers is not satisfactory.

### Overfitting

As mentioned above, multilayer networks are capable of performing just about any linear or nonlinear computation, and they can approximate any reasonable function arbitrarily well. However, while the network being trained might theoretically be capable of performing correctly, backpropagation and its variations might not always find a solution [30, 31, 33, 37]. Fortunately, this was not the case with any of the ANN architectures that were tested in this study.

Important also is the linearity of the network. The error surface of a nonlinear network is more complex than the error surface of a linear network. Nonlinear transfer functions in multilayer networks introduce many local minima in the error surface [33, 37]. As gradient descent is performed on the error surface, depending on the initial starting conditions, it is possible for the network solution to become trapped in one of these local minima. Settling in a local minimum can be good or bad depending on how close the local minimum is to the global minimum and how low an error is required. The NN user should be cautioned that although a multilayer backpropagation network with enough neurons can implement just about any function, backpropagation does not always find the weights for the optimum solution. One might need to reinitialize the network and retrain several times, in order to reach the best solution. Fortunately, in this study, there is evidence that the minima of the performance function (cross-entropy or mse) found during each NN training cycle, were of a global type, rather than being local minima that would have brought significant variation in the estimated weights and biases, and in the final results in terms of sensitivity and specificity.

The partitioning of the data set (70% of the data randomly assigned to training, 15% of the data randomly assigned to validation, and 15% of the data randomly assigned to the test set) works well for larger data sets such as ours, and is widely used in ANN applications reported by other authors (with small deviations). Here, during training, the cross-entropy error reached its minimum after just three iterations, which means that there were enough data in the training set to achieve good performance without underfitting, i.e. no need to dedicate more data to the training set. The error started to increase at iteration number 26, as a sign of overfitting starting to occur, which is when the training was automatically stopped. This is a sound training behavior, and there was no apparent reason to reallocate the data. Moreover, during testing, all three data subsets reached their minimum simultaneously (Fig. 3). As mentioned above, if the error in the test set reaches a minimum at a significantly different iteration number than the validation set error, this might indicate a poor division of the data set [43]. Since this did not happen, there was no reason to consider data reallocation. The test subset, as a set of examples used to assess the performance of the fully-trained classifier, did not differ significantly in performance from the validation subset, which was another indication of proper division. It should also me mentioned that the test set is important when comparing different models (in terms of number of hidden layers, number of neurons in a layer, etc.). For this reason, it should not be chosen to be smaller than the 15% used here.

The issue of overfitting was addressed in the Methods section, under Generalization. Many other methods have lately been proposed to improve generalization. For example, in [44], the authors employed a technique called “dropout”, to address this problem. The key idea is to randomly drop units (along with their connections) from the neural network during training. This prevents units from co-adapting too much. During training, samples from an exponential number of different “thinned” networks are dropped out. At test time, it is easy to approximate the effect of averaging the predictions of all these thinned networks by simply using a single unthinned network that has smaller weights. This significantly reduces overfitting and gives major improvements over other regularization methods.

### Advantages and disadvantages of using ANN versus statistical methods

The artificial neural networks have evolved as an alternative to classical statistical methods for classification, such as discriminant analysis, or for developing predictive models for dichotomous outcomes in medicine. They offer many advantages, including less formal statistical training and the ability to implicitly detect complex non-linear relationships. Disadvantages include proneness to overfitting and the empirical nature of model development [45]. It is unlikely that one of the above methods will be the technique of choice in all circumstances. The choice as to which technique should be used depends to a large extent on the nature of a particular data set. For example, logistic regression and discriminant analysis are believed to be the more appropriate choice when the primary goal of model development is to look for possible causal relationships between the independent (input) and dependent (output) variables, and the modeler wishes to easily understand the effect of predictor variables on the outcome. Neural networks appear to be particularly useful when the primary goal is outcome prediction or pattern recognition/classification, and important interactions or complex nonlinearities exist in the data set [45].

The transparency of the decision making process has always been an issue in diagnostic decision making. Undoubtedly, it would be advantageous to be able to trace the logical flow at every step of the way, as was done with the “expert systems” of the 80s. Yet, they did not become a major trend. In a multifactor diagnostic system, it is increasingly difficult to follow the contribution of each and every factor. Which is how factor analysis, logistic regression, discriminant analysis, principal component analysis and of course neural network emerged. In fact, in [18], we used linear discriminant functions for decision making. Their use is relatively simple during the decision making, but derivation based on the training set is not simpler than the backpropagation method used here for ANN. And just as it may be uncertain to go beyond linear discriminant functions (e.g. use higher order discriminant functions such as quadratic functions) potentially causing overfitting in discriminant analysis, it is risky to “overtrain” an ANN. Yet, there is one difference: it is usually simpler to modify the structure of the ANN (oftentimes empirically), than that of the discriminant function model.

Last but not least, in a way, one can reduce an ANN to a logical structure which is identical to a linear discriminant function, in our case, use just one of the four neurons in the hidden layer, and use linear functions for f^1^ and f^2^, instead of log-sigmoid functions (*logsig*), as in this work (vector **LW** will, of course, turn into a scalar). In fact, it is the increased complexity that brings about the potentially improved functionality of ANNs.

## Conclusions

This study confirmed that spectral powers at several signal frequencies obtained with retinal birefringence scanning around the human fovea can be used to detect central fixation reliably. Artificial neural networks can be trained to deliver very high diagnostic precision which is at least as good as statistical methods. In our case, ANN precision turned out to be even slightly better than the precision achieved with all discriminant analysis based methods, albeit with a relatively small size of the training set. It will take a larger training set to prove definite improvement. Although the ANN method was applied to one specific optical instrument design (spinning wave plate), there is enough evidence that neural-networks-based classifiers will work with other optical designs, producing other frequencies and combinations thereof. Regardless of the relatively small initial sample size, we believe that the PVS instrument design, the analysis methods employed, and the device as a whole, will prove valuable for mass screening of children. The instrument robustly identifies eye misalignment, which is a major risk factor for amblyopia, and the addition of a neural network based diagnostic feature will undoubtedly improve its performance.

## Abbreviations

ANN: artificial neural network
BP: backpropagation method for training the neural network
CAL: calibration data
CF: central fixation
FNN: feedforward neural network
HWP: half wave plate
FN: false-negative
FP: false positive
IRB: institutional review board
IW: input weight matrix
LM: Levenberg–Marquardt training algorithm
LW: output weights matrix/vector
mse: mean squared-error
NN: neural network(s)
para-CF: paracentral fixation (off-central fixation)
PVS: pediatric vision screener
PhSS: phase-shift-subtraction
RBS: retinal birefringence scanning, retinal birefringence scanner
SBJ: clinical data
SNS: sensitivity
SPC: specificity
TN: true negative
TP: true positive
WP: wave plate
